# Elevation of Inducible Nitric Oxide Synthase and Cyclooxygenase-2 Expression in the Mouse Brain after Chronic Nonylphenol Exposure

**DOI:** 10.3390/ijms9101977

**Published:** 2008-10-24

**Authors:** Yan-Qiu Zhang, Zhen Mao, Yuan-Lin Zheng, Bao-Ping Han, Ling-Tong Chen, Jing Li, Fei Li

**Affiliations:** 1 School of Environment Science and Spatial Informatics, China University of Mining and Technology, Xuzhou 221008, Jiangsu Province, P.R. China; 2 Key Laboratory for Biotechnology on Medicinal Plants of Jiangsu Province, School of Life Science, Xuzhou Normal University, Xuzhou 221116, Jiangsu Province, P.R. China

**Keywords:** Nonylphenol, iNOS, COX-2, NO, brain

## Abstract

The present study was performed to investigate the effects of chronic administration of nonylphenol (NP) on the expression of inflammation-related genes in the brains of mice. NP was given orally by gavages at 0, 50, 100, and 200 mg/kg/d. The expression of inflammatory enzymes, inducible nitric oxide synthase (iNOS) and cyclooxygenase-2 (COX-2), was evaluated by immunohistochemistry and immunoblotting assays. The nitric oxide (NO) level and nitric oxide synthase (NOS) activity were also measured by biochemical analyses. The results showed that NP at a high dose (200 mg/kg/d) significantly increased the expression of iNOS and COX-2 in both the hippocampus and cortex. In parallel with the increase in iNOS expression, the NO level was significantly greater at the dose of 200 mg/kg/d, compared to the control. The activity of NOS was also increased in the brain of mice at the dose of 100 and 200 mg/kg/d. These findings demonstrate that NP may have the potential to induce the chronic inflammation or cause neurotoxicity in the mouse brain.

## 1. Introduction

Nonylphenol (NP), a major degradation product of alkylphenol ethoxylates, is wildly used in the synthesis of detergents and as an antioxidant. Accumulating data suggested that NP, as an endocrine disrupter, has adverse effects on the reproductive and immune systems. *In vivo* studies have indicated that NP can exert a generally chronic toxicity to epididymis, induce apoptosis of testicular cells, and indirectly disturb the secretions of hormones and the production of sperm [1]. NP with estrogen-like activity might affect the regulation of the immune function through thymocyte apoptosis [2]. In recent years, evidence indicates that NP showed neurotoxicity in the central nervous system [3–5]. But so far, the precise mechanisms behind the neurotoxicity of NP have not been elucidated.

Inflammation is the body’s normal protective response to an injury, irritation or surgery. It can be acute or chronic. When acute, it occurs as an immediate response to trauma. When it is chronic, the inflammation reflects an ongoing response to a longer-term medical condition. Chronic inflammation can also be from unresolved inflammation [6]. Unresolved inflammation, whether due to infection, autoimmunity or environmental agents, markedly increases the risk of cancer [7]. Uncontrolled chronic inflammation in the central nervous system (CNS) can lead to neurodegenerative disorders like Alzheimer’s disease (AD) [8] and Parkinson’s disease [9]. Inflammation is also detrimental for neurogenesis in adult brain [10].

Although the precise mechanism behind chronic inflammation has not been elucidated, increasing evidence has suggested that pro-inflammatory cytokines, chemokines, adhesion molecules and inflammatory enzymes are involved in chronic inflammation. Nitric oxide synthase (NOS) is an enzyme in the body that contributes to transmission from one neuron to another, to the immune system and to dilating blood vessels. NOS catalyzes the conversion of L-arginine to nitric oxid (NO), which is related to neuronal function and neurotransmission. There are three forms of NOS in the CNS: the neuronal form (nNOS), the endothelial form (eNOS), and an inducible form (iNOS) [11]. Aberrantly expressed iNOS and elevated levels of NO considered the most important neurotoxic effectors during AD [12].

Cyclooxygenase (COX) is the enzyme involved in the production of prostanoids from arachidonic acid. Cyclooxygenase plays a key role in inflammation and fundamental brain functions. Two isoforms of COX have been identified: COX-1 is a constitutive isoform with housekeeping functions, whereas COX-2 is an inducible isoform in most tissues. But in brain COX-2 is also constitutively expressed and involves in brain functions [13]. Much evidence suggests that the expression of COX-2 is elevated in AD brain, and COX-2 has been explored as a therapeutic target for anti-inflammatory treatments [8, 14].

To extend our understanding of the effects of NP on nervous system, in the present study we have evaluated the expression of COX-2 and iNOS, and also the NO level and the activity of NOS *in vivo*. These data provide a first view of the inflammatory, neurotoxic effects of NP.

## 2. Results and Discussion

The central nervous system is highly sensitive to exogenous chemicals. Exogenous chemicals such as endocrine disrupters may break homeostasis and affect normal brain functions [15, 16]. *In vivo* studies on the metabolism and organ distribution of NP in juvenile salmon suggested that NP is able to penetrate the blood-brain barrier and hence may have effects on the fish hypothalamus-pituitary axis [17]. When NP was administered by gavage to pregnant rats it could also cross the placental barrier, change fetal blood-brain permeability and reach the brain [18]. A metabolic balance study showed that residual concentrations of NP calculated in samples from rats fed a dose of 10 mg/kg were about 10,000-fold higher than those recorded in rats fed a dose of 1 μg/kg [19]. This indicated that NP could reach the brain and may accumulate in it for a short time. Evidence suggests that NP has adverse effects in the nervous system [3–5]. However, the precise mechanisms behind the neurotoxicity of NP have not been elucidated. To extend our understanding of the effects of NP in the nervous system we evaluated the expression of COX-2 and iNOS, and also the activity of NOS and the NO level *in vivo*.

The no-observed-adverse-effect level (NOAEL) of NP on reproductive capacity is 50 mg/kg/d, or greater, in parent rat [20]. In Han’s study, NP at 250 mg/kg/d could exert a generally chronic toxicity to rats and a particular toxicity to epididymis and can induce the apoptosis of testicular cells [1]. So we selected the lower dose of 50 mg/kg/d and the higher dose of 200 mg/kg/d for use in this study, which is close to the NOAEL of rats but lower than the maximum tolerated dose that could be used without signs of overt toxicity. After the administration of NP for 90 days, the mice used in this experiment all are in good health, and no mortality was observed at the highest doses.

### 2.1. NP increases the NO level in the mice brain

**Figure 1.**
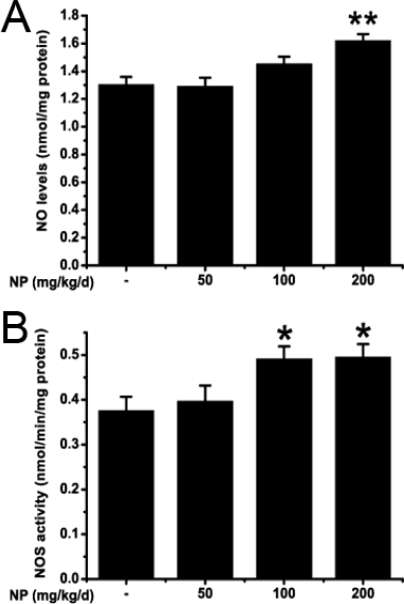
The effect of NP on NO level (A) and NOS activity (B) in mouse brain. Values are expressed as mean ± S.E.M. (n = 9). *P < 0.05; **P < 0.01 vs. the vehicle control.

Biochemical analysis was used to detect the NO levels in the mouse brain. The results showed that NP could significantly increase the NO levels in the brain (F3, 32 = 8.409; P = 0.000). NO levels were elevated at the dose of 200 mg/kg/d (P = 0.001) but no changes were observed at dose of 50 and 100 mg/kg/d (50 mg/kg/d: P = 0.998; 100 mg/kg/d: P = 0.213) (Figure 1A). NO is a pleiotropic free radical that regulates various physiological functions in the brain [21]. The proper amount of NO induces vasodilatation, inhibits apoptosis and plays an important role in memory processes. However, when NO is produced in an excessive amount it can be harmful, mainly under oxidative stress conditions, due to the oxidation and nitrotyrosination of functional proteins [22]. Our results showed that high dose of NP could increase NO production in the mouse brain. NO is one of a reactive nitrogen species. It is also easily converted into peroxynitrite anion, which is highly reactive and can cause cytotoxic radical chain reactions[23, 24, 25]. In the presence of carbon dioxide, peroxynitrite can readily modify proteins by forming nitrotyrosine, one of the markers for oxidative injury[26], and the presence of nitrotyrosine will be detected in our future research. NO caused oxidative stress plays a role in all neurodegenerative processes, such as Parkinson’s disease (PD), Huntington’s disease (HD), Alzheimer’s disease (AD), amyotrophic lateral sclerosis (ALS), multiple sclerosis (MS) and ischemia [22]. Despite the oxidation, overproduction and accumulation of NO can initiate apoptosis which is also involved in the NO induced neurotoxicity [25, 27, 28].

### 2.2. NP increases the NOS activity and the iNOS expression in the mice brain

**Figure 2.**
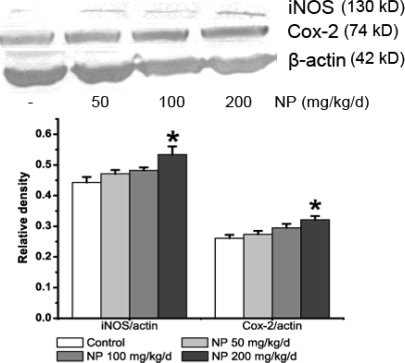
Western blotting analysis of the expression of iNOS (130 kD) and COX-2 (74 kD) in the brains of control mice and NP-treated mice. The relative ratio of colorimetric density of iNOS/β-actin and COX-2/β-actin was analyzed by Quantity one (Bio-Rad, USA). β-actin was used as an internal control. All experiments were carried out at least in duplicate on three different animals and values are expressed as mean ± S.E.M. (n = 3) *P < 0.05 vs. the vehicle control.

**Figure 3.**
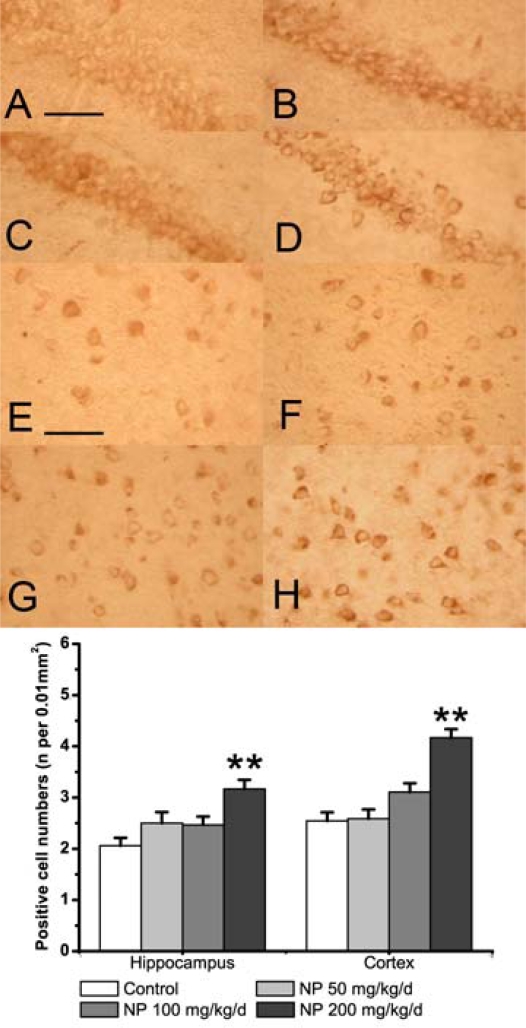
The effect of NP on the expression of iNOS as determined by immunohistochemistry in the hippocampus (A-D) and cortex (E-H). (A, E) Vehicle control. (B, F) NP 50 mg/mL per day group. (C, G) NP 100 mg/mL per day group. (D, F) NP 200 mg/mL per day group. Scale bars (A–H): 100 μm. The bands below show the number of iNOS positive cells in the hippocampus and cortex. Values are expressed as mean ± S.E.M. (n = 9). **P < 0.01 vs. the vehicle control.

To explore the reason for NO increase we measured the activity of NOS and the expression of iNOS in the mouse brain. NOS activity was induced by the treatment of NP (F3, 32 = 4.307; P = 0.012), especially at 100 and 200 mg/kg/d (100 mg/kg/d: P = 0.048; 200 mg/kg/d: P = 0.040), whereas the NOS activity at the dose of 50 mg/kg/d was not different from the activity observed in control mice (P = 0.096) (Figure 1B). iNOS expression was determined by immunoblotting assays and immunohistochemistry. As shown in the result of western blot (Figure 2) a band at the position of the 130 kDa protein was exhibited in protein extracts from the whole brain. Exposure of NP induced a concentration-dependent increase of iNOS protein expression (F3, 8 = 4.801; P = 0.034). In particular, NP at the dose of 200 mg/kg markedly increased the iNOS protein expression (P = 0.025) (Figure 2). Immunohistochemistry was performed to further explore the distribution and cellular localization of iNOS immunoreactivity in the hippocampus and cortex (Figure 3). A low level of basal iNOS immunoreactivity was present in both the hippocampus and the cortex of control mice. The number iNOS-positive cells in the cortex was significantly increased at the dose of 200 mg/kg/d (P < 0.01). There was no significant change at the dose of 50 and 100 mg/kg/d (P > 0.05) (Figure 3). Similarly, the numbers of iNOS positive neurons were significantly increased in the hippocampus of 200 mg/kg/d NP (P < 0.01) dosed mice only. This suggests that treatment with NP at a higher dose (200 mg/kg/d) clearly causes a significant increase in the numbers of iNOS-positive cells in both the hippocampus and the cortex.

iNOS is a crucial enzyme that participates in chronic inflammation of the CNS. iNOS is usually expressed after inflammatory, neurodegenerative or ischemic brain damage in CNS [29]. The up-regulation of iNOS produces an ample supply of NO, which can cause neurotoxicity. In our study, the activity of NOS at 100 and 200 mg/kg/d was higher than those in the control, and the expression of iNOS was also significantly increased in hippocampus and cortex of the NP treated mice. Other exogenous chemicals could likewise regulate the iNOS expression. Subchronic administration of 3-monochloro-1,2-propanediol (3-MCPD) increased iNOS expression in the rat brain [11]. 1,1,1-Trichloro-2-(*p*-chlorophenyl)-2-(*o*-chlorophenyl)ethane (o,p’-DDT) also increased iNOS and proinflammatory cytokines expression levels in mouse macrophages[30]. The augmented iNOS expression and NOS activity could lead to increase NO production in the mouse brain. Over-expression of NO is toxic to the CNS. So, the NP-induced neurotoxicity is mediated, at least in part, through disturbances in the nitric oxide signaling pathway.

### 2.3. NP increases the expression of COX-2 in the mice brain

**Figure 4.**
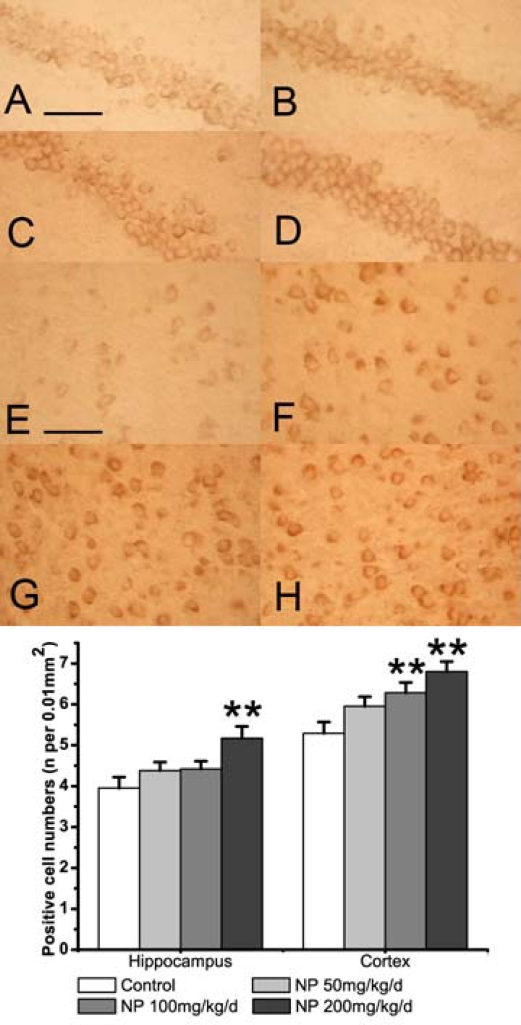
The effect of NP on the expression of COX-2 as determined by immunohistochemistry in the hippocampus (A-D) and cortex (E-H). (A, E) Vehicle control. (B, F) NP 50 mg/mL per day group. (C, G) NP 100 mg/mL per day group. (D, F) NP 200 mg/mL per day group. Scale bars (A–H): 100 μm. The bands below show the number of COX-2 positive cells in the hippocampus and cortex. Values are expressed as mean ± S.E.M. (n = 9). **P < 0.01 vs. the vehicle control.

The effect of NP on the expression of COX-2, anther pivotal inflammatory enzyme, was also detected in the mouse brain. The result of immunoblot analysis showed a single band at the position of a 74 kDa protein (Figure 2). Chronic NP treated at the higher dose (200 mg/kg/d) enhanced the expression of COX-2 (P < 0.05). The results of the immunohistochemical analysis were consistent with the immunoblot data. COX-2 immunoreactivity was present in all of the studied areas (Figure 4). In the cortex the number of COX-2 positive cells significantly increased after treatment of NP at 100 or 200 mg/kg/d dosing compared with the control group (P < 0.01). In hippocampus the COX-2 immunoreactivity occurs in neurons of the pyramidal cell and granule cell layers. Treatment with NP increased the numbers of COX-2 positive cells, especially at the highest 200 mg/kg/d dose (P < 0.01). These data showed that treatment with NP at a higher dose (200 mg/kg/d) significantly increased the numbers of COX-2 positive cells in both the cortex and the hippocampus.

COX-2 is present in the perinuclear, dendritic, and axonal areas of glutamatergic neurons, particularly in the cortex, hippocampus, and amygdala [31]. COX-2 is expressed under normal conditions and contributes to fundamental brain functions, such as synaptic activity, memory consolidation, and functional hyperemia [32]. Overexpression of COX-2 could contribute to the enhancing effect of PGE2 on glutamate release, and the oxidative stress-mediated damage, by producing oxidizing reactive species [32]. In addition, COX-2 is involved in the formation of the amyloid plaques through the increased of Aβ in AD [33]. Our experiment indicated that higher dose of NP stimulate the expression of COX-2. A persistent overexpression of COX-2 would trigger the chronic inflammation in the CNS, which could lead to neurodegenerative disorders. As the expression of COX-2 increased in brain areas related to memory (the cortex and the hippocampus), this raised the suspicion of possible effects of NP on learning and memory.

In summary, these results demonstrate that the endocrine disruptor NP causes marked expression of COX-2 and iNOS in the mouse brain, suggesting that inflammation might be involved in the NP-induced neurotoxicity in the brain.

## 3. Experimental Section

### 3.1. Animals and treatments

Thirty-six young male (Kun Ming) mice (4-week-old and with weight 26.90 ± 3.29 g, purchased from the Branch of National Breeder Center of Rodents, Shanghai, P.R. China) were randomly divided into four groups. Nine mice were housed per cage on a 12 h light/dark cycle, with *ad libitum* access to food and water. After an adaptation period, nonylphenol (a mixture of branched side chains containing 85% *p*-isomers, Fluka, Buchs, Switzerland) was dissolved in corn oil and administered orally at 50, 100 or 200 mg/kg per day for 90 days. Mice in the control group were given the vehicle (corn oil) alone. All experiments were carried out in accordance with the Chinese legislation on the use and care of laboratory animals and were approved by the respective university committees for animal experiments. All efforts were made to minimize suffering of experimental animals during all procedures. The animals were sacrificed 24 hours after the last administration of NP. The brain tissues were rapidly removed from the skulls, frozen immediately in liquid nitrogen and stored at −70 °C until analysis.

### 3.2. Brain nitrates and nitrites (NOx) assay

Brain nitrates and nitrites (NOx) content was estimated by using the method of Tracey *et al*. [34]. Brain samples were homogenized in distilled water and centrifuged at 10,000×g for 15 min at 4 °C. The brain homogenates were treated with nitrate reductase +NADPH+FAD, and incubated for 1 h at 37 °C in the dark. Then ZnSO_4_ was added to precipitate the proteins. After centrifuging at 6,000×g, the supernatants were added with Griess reagent (1:1 mixture of 1% sulphanilamide in 5% H_3_PO_4_ and 0.1% *N*-(1-naphthyl)ethylenediamine) for color development. The plates were then read at 540 nm, and NOx was calculated by using a nitrate standard curve.

### 3.3. Assay of Nitric oxide synthase activity

NOS activity was inferred by measuring total nitrite concentration and subsequent colorimetric assay[35]. Tissue samples were mixed with the assay solution contained 0.1 mmol/L L-arginine, 1 μmol/L NADPH, 1μmol/L cofactors (FADH, FMNH and H_4_B), 0.5 mmol/L EDTA, 1.2 mmol/L CaCl_2_, 0.5 mmol/L MgCl_2_, 0.2 U nitrate reductase, 0.5 mmol/L glucose-6-phosphate, 0.4 U glucose-6-phosphate dehydrogenase Then the mixtures were incubated for 45 min at 37 °C. After incubation for 10 min with Griess reagent, the amount of reaction product was estimated the absorbance at 540 nm. Protein concentration was determined by using the BCA assay kit (Pierce Biotechnology Inc., Rockford, IL) with bovine serum albumin (BSA) used as standard.

### 3.4. Western blot

For Western blot analysis, the brain of each animal was homogenized separately in 10 mL/g wet weight of cold homogenization buffer containing 50 mM Tris–HCl (pH7.4), 150 mM NaCl, 1% NP-40, 0.1% SDS, 5 mM sodium fluoride, and Protease Inhibitor Cocktails (Sigma). The homogenate was then centrifuged at 10,000×g for 20 min at 4 °C, and the supernatants were collected. Protein concentrations were measured using BCA assay (Pierce). Proteins were separated on 12% sodium dodecyl sulfate–polyacrylamide gels with 4% stacking gel and transferred to PVDF Western blotting Membrane (Roche). After blocking with 5% w/v BSA in TBS containing 0.1% Tween 20, the membranes were then incubated overnight at 4 °C with anti-iNOS (1:5,000, Chemicon) or anti-COX-2 (1:1,000, Cell Signaling) antibody. Subsequently membranes were incubated for 1 h at room temperature with Anti-rabbit-IgG HRP-linked Antibody (Cell Signaling Technology, Inc.) secondary antibody. Immunoreactivity was visualized using Stabilized TMB Substrate for Horseradish Peroxidase (Promaga). Band intensity was analyzed by gel documentation system (BioRad). For all western blot analysis, β-actin (1:5,000, Chemicon) was used as the loading control. The protein levels were expressed as densitometry (% of the loading controls).

### 3.5. Immunohistochemistry

Immunohistochemistry was performed on one set of 12 μm cryostat sections. After blocking with 5% BSA, The sections were incubated with anti-iNOS (1:5,000, Chemicon) or anti-COX-2 (1:1,000, Cell Signaling) antibody overnight at 4 °C. Then the samples were exposed to biotinylated goat anti-rabbit IgG secondary antibody (Vector Laboratories Inc., Burlingame, CA, USA) at 1:1000 dilution for 1 h at room temperature. The immunoreactive cells were visualized with diaminobenzidine (Sigma) by light microscopy. The number of positive cells in 0.01 mm^2^ was estimated by blind manual counting of seven regions located at a consistent position per section.

### 3.6. Statistical analysis

Results were shown as the mean ± S.E. Statistical significance between groups was analyzed by one-way analysis of variance (ANOVA) using post-hoc Tukey’s test. P-values less than 0.05 were considered significant.
